# Junctionless Silicon Nanowire Transistors without the Use of Impurity Doping

**DOI:** 10.1021/acsnano.5c17282

**Published:** 2026-02-23

**Authors:** Soundarya Nagarajan, Dirk König, Ingmar Ratschinski, Giulio Galderisi, Somayeh Shams, Thomas Mikolajick, Daniel Hiller, Jens Trommer

**Affiliations:** † 435872 NaMLab gGmbH, Nöthnitzer Str. 64a, Dresden 01187, Germany; ‡ Institute of Semiconductors and Microsystems, Technische Universität Dresden, Nöthnitzer Str. 64, Dresden 01187, Germany; § Department of Material Physics, Research School of Physics, 2219 Australian National University (ANU), Canberra ACT 2601, Australia; ∥ Institute of Applied Physics (IAP), Technische Universität Bergakademie Freiberg, Leipziger Str. 23, Freiberg 09599, Germany

**Keywords:** junctionless transistor, silicon transistor, direct modulation doping, cryogenic electronics, charge carrier transport

## Abstract

With the shrinking dimensions of semiconductor structures reaching the nanoscale, conventional impurity doping techniques face several challenges due to their statistical nature, temperature dependence, and degradation in efficiency of the doping method. In addition, the cryogenic operation of highly doped transistors is complicated due to carrier freeze-out, which significantly reduces the availability of mobile charges, degrading device performance and inducing noise. Here, an innovative material solution is presented that enables silicon nanowire junctionless transistors without requiring impurity doping within the active semiconductor region. To this end, a SiO_2_ dielectric shell with deliberate defect engineering surrounding both the channel and the contact regions - known as direct modulation doping—is used to modify the nanoscale transport properties of the silicon. The obtained active carrier densities in the experiment are comparable to highly impurity-doped devices in the range of $∼10^{18}cm^{-3}$ and remain stable over a broad temperature range from 400 K down to 77 K. The primary advantage of removing dopants from the channel is evident in the enhanced field-effect mobilities, which increase from 115 to 331 cm^2^V^–1^s^–1^ as temperature decreases. The fabricated nanowire transistors in this work provide a high on/off ratio of ≥10^6^, and a stable on-state performance down to 77 K. Hybrid-density-functional-theory calculations are carried out to show that there are no fundamental roadblocks to employing the method to devices with ultrascaled dimensions. The device architecture is positioned for applications in energy-efficient cryo-electronics and quantum technologies by addressing the limitations associated with conventional impurity doping.

## Graphical abstract

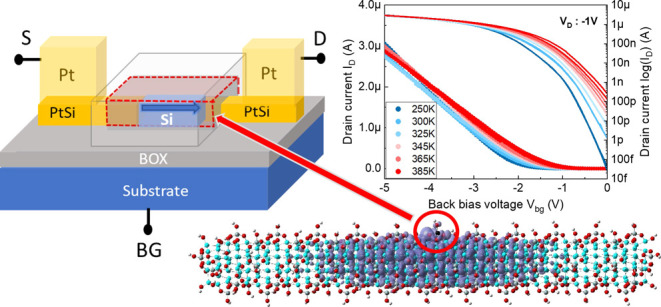

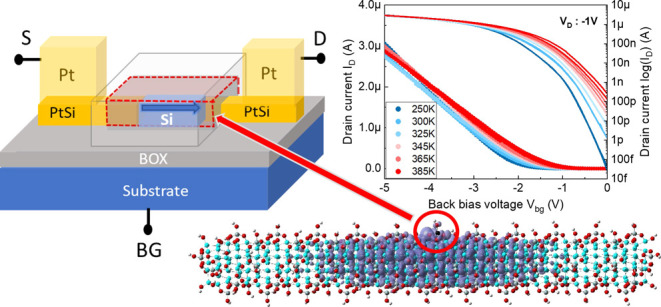

## Introduction

Impurity doping is an elementary process to tune the conductivity of silicon. However, with continuous miniaturization of semiconductor nanostructures, challenges related to the impurity doping process^1–4^ such as increased ionization energy, dopant deactivation, dopant segregation, impurity scattering, random dopant fluctuation and out-diffusion have a severe impact on a variety of nanotechnological solutions requiring well-defined carrier transport properties, ranging from cryogenic electronics,^5^ to neural interfaces^6,7^ and solid-state batteries.^8^ A device concept suffering in particular from such shortfalls is the Junctionless field-effect transistors (JLFETs).^9^ Unlike traditional transistors, where the current flow is controlled by gated p–n junctions, JLFETs^10,11^ operate with a uniformly doped channel typically requiring a high impurity concentration in the range of 10^18^–10^19^ cm^–3^. Especially at cryogenic temperatures,^12,13^ the impurity scattering induced mobility degradation and noise related to these defects subjects the device to considerable performance penalties for application scenarios like qubit control electronics^14,15^ or cold data centers.^16^ Ultimately, an alternate technique that removes the difficulties of doping at the nanoscale is crucial. Charge transfer doping has emerged as an efficient method for semiconductor nanostructures to tune the transport properties by charge transfer at the interface with surface dopants.^17^ The energy-level alignment of the surface dopants with respect to the semiconductor’s band edges favors the generation of holes or electrons as carriers in bulk. Although this technique is widely exploited for graphene, MoS_2_, carbon nanotubes, and a few other 2D host materials,^18–20^ its application has not been extensively studied in silicon.

In this work, we report an alternative technique that allows for the operation of a p-type silicon junctionless transistor where the channel is no longer doped, thereby resolving many drawbacks traditionally associated with the JLFET concept, such as random dopant fluctuations and mobility degradation. Thereto, defect-engineered impurities are moved into an ultrathin silicon dioxide (SiO_2_) dielectric layer around the channel. This SiO_2_ layer is capable of providing free holes as majority carriers to silicon by means of a charge transfer mechanism between the acceptor impurities (aluminum-Al) in SiO_2_ and the adjacent silicon volume. The energy level of the trivalent Al acceptors in SiO_2_ is ca. 0.5 eV below the Si valence band edge (VBE),^21^ and provides the relaxation energy for transferring electrons from the valence band (VB) of silicon, thereby generating a high density of free holes in silicon. Thus, the silicon becomes strongly p-type in the process. As a result, the controlled p-doping in SiO_2_ determines the position of the Fermi level in silicon.^21,22^ This approach, known as direct modulation doping, was predicted by density-functional theory (DFT) calculations, and later confirmed in experiments by capacitance–voltage measurements and deep-level transient spectroscopy (DLTS).^21^ Direct modulation doping can effectively adjust the electronic properties of the channel, although being spatially separated from the semiconductor material. Furthermore, the ionized Al acceptors in the SiO_2_ dielectric represent a significant amount of fixed charges that induce a strong drift field, thereby bringing about a strong upward band bending in silicon at the Si/SiO_2_ interface.^21–23^ This field continues into the SiO_2_ layer with modulation acceptors and prevents all acceptors from being ionized by an interdopant Coulomb blockade, resulting in a self-regulation of the doping mechanism (see Supporting Information).^24^ The associated existence of a two-dimensional hole gas at the modulation-doped silicon surface was indicated by optical methods.^25^ While such works provided an indirect proof of the electron capture mechanism from silicon, direct evidence that Al acceptor modulation doping significantly modifies the conductivity of silicon has been demonstrated through transport measurements in Si nanochannels.^26–28^ This doping method also has a high spatial selectivity and the ability to adjust the density of carriers induced in silicon. Recently, a nearly constant sheet carrier density of $∼4.7\times 10^{12}cm^{-2}$ measured from 300 K down to 2 K and a mobility improvement from 89 cm^2^V^–1^s^–1^ at 300 K to 227 cm^2^V^–1^s^–1^ at 10 K was demonstrated by Hall effect measurements on modulation-doped Si Hall-bar structures.^5^ While the measurements on these test structures imply a high potential for operation even at extreme cryogenic temperatures, no functional electrical device has been created based on direct modulation doping so far.

In this study, modulation doping of a SiO_2_ shell is employed to demonstrate silicon junctionless transistors without classical doping of the silicon active channel region, demonstrating the effectiveness of this method to replace conventional impurity doping and enabling reliable operation at cryogenic temperatures. The proposed doping method is first modeled via hybrid density function theory (h-DFT) calculations to comprehend the core physical principles in quantitative detail, and elucidate on the spatial-density relationship of the modulation doping impact at the lower end of structure size where direct modulation doping is still applicable. Subsequently, a p-type operation of a junctionless field-effect transistor is demonstrated experimentally for devices with a single nanowire (NW) and an array consisting of ten NWs in parallel. The device characteristics are also studied down to a temperature of 77 K. Next, the carrier transport properties are studied in two-ways: by characterizing the transport mechanism across the contact interface by evaluating the temperature behavior of the device from 250 K up to 385 K and by analyzing the carrier density and field-effect mobility over a broad temperature range from 400 K down to 77 K using the gated transfer length method (TLM) approach on specialized test structures. The methods section describes the detailed fabrication procedure and gives an overview of the measurement setup. Supporting Information (Figure S1) further elaborates on a schematic overview of the fabrication process.

## Results and Discussion

### Modulation Acceptor Doping for Silicon Nanowire Junctionless Transistor

To design our experiments, modulation acceptor doping of silicon nanowires with SiO_2_ shell was modeled by DFT to gain an in-depth understanding of the underlying mechanism governing charge transport and assess the impact of extreme dimensional scaling of nanowire architecture on the electronic properties. Real space (nonperiodic boundary conditions) h-DFT calculations were carried out on a Si NW embedded in 2 atomic monolayers (MLs) of SiO_2_. This NW consists of 395 Si atoms (917 atoms total), is 1.2 nm thick, and 8.8 nm long, and extends as far toward a device-relevant structure, e.g., a gate-all-around (GAA)-NW FET, as the size limit allowed by the DFT approach under the chosen computational route permits.^30^ Indeed, we employed our own add-on code to scale up the size limit typically given by the Gaussian_09 program package^31^ by more than a factor of 2. More details on the exact implementation are given in the methods section. Figure 1 shows the electronic properties of the Si nanowire with relevance to the modulation acceptor (MA) state in SiO_2_. As expected,^21,24^ the free hole associated with the MA state is dispersed widely throughout the NW (Figure 1e), whereby its density is highest at the MA (Figure 1c). Supporting Information S2 refer to a more detailed video illustration the evolution of the spatial distribution of the hole charge density associated with the direct modulation acceptor states added to the SiO_2_. Figure 1b shows that the MA energy level *E*_MA_ is located above the valence band energy level (*E*_V_) of the NW in analogy to an impurity acceptor in Si, which appears to contradict experimental findings.^24^ However, in contrast to this previous experiment, the simulated NW is ultrasmall with a bandgap of *E*_gap_ = 1.67 eV. The associated strong quantum confinement (QC) pushes EV of the NW significantly further below the vacuum energy level *E*_vac_, whereby *E*_V_ passes *E*_MA_ in its binding energy. Interestingly, these DFT results show that free holes still could be delivered to Si-based systems with substantial QC, albeit requiring a thermal ionization energy *k*_B_T as is required for an impurity dopant. All other detrimental properties of impurity dopants in Si are still eliminated with the direct MA in SiO_2_. The NW dimensions of 1.2 nm diameter and 8.8 nm length thus define the minimum spatial extension of a Si nanovolume which still can be doped by an MA state in SiO_2_. The mere 1 ML SiO_2_ separating the MA from the Si NW shows that such MAs in SiO_2_ are extremely robust. Thereby, ultrathin tunneling layers can be implemented which still uphold the quantum-chemical environment for the Al-induced MA to yield a free hole to adjacent Si. Such ultrathin layers are also of important for hole-selective contacts at Si solar.^21,32^ For VLSI devices, this immediate proximity of MA states in SiO_2_ to Si allows for a maximum relaxation energy *E*_relax,MA_ = *E*_V_(Si) – *E*_MA_(SiO_2_). Since the degree of MA ionization and thereby the areal hole density delivered to Si depends on the equilibrium of *E*_relax,MA_ and the Coulomb blockade between negatively ionized MA states *E*_Coul,MA_ ^24^ the minimum MA distance in SiO_2_ from Si of 1 ML yields the maximum hole density deliverable to Si. With the virtually nonexistent thermal diffusion of Al atoms within SiO_2_ even at *T* = 1000 °C over 1 h^21^ a tunneling layer of 1 to 2 ML SiO_2_ obtains technological significance for applications such as ultimate miniaturization for very-large-scale-integration, but also hole selective contacts in Si solar cells. Supporting experimental results on the tunneling interface thickness design on a MOS reference capacitor can be found in the Supporting Information. The analysis reveals that there are no general roadblocks to employing this doping technique to devices with ultrascaled dimensions.

**Figure 1:**
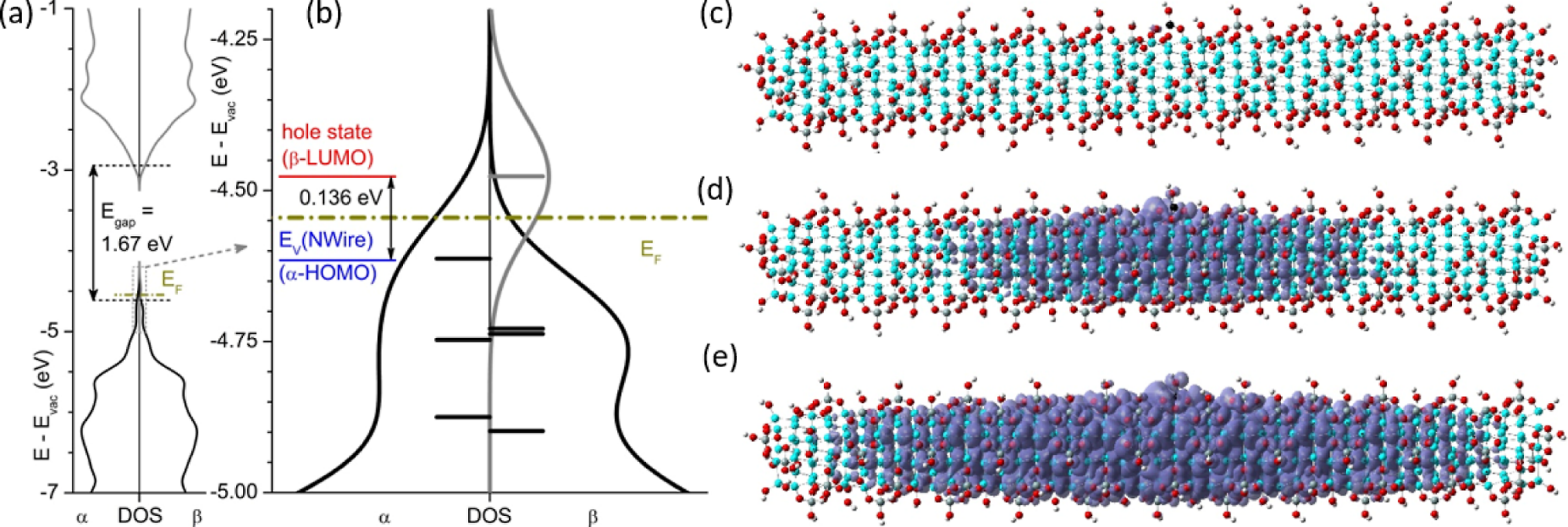
DFT results of 1.2 nm thick and 8.8 nm long Si NW in 2 ML SiO_2_ with one Al–O-DB modulation acceptor (MA) complex. (a) Electronic density of states (DOS) of the entire NW in the energy region relevant for electronic states of the Si NW. Binding energies are shown relative to the vacuum energy level *E*_vac_, occupied (unoccupied) electronic DOS are shown by black (gray) lines. Due to the MA state breaking spin parity, the electronic DOS is shown for the two possible electron spin configurations α and β. (b) Detail of frontier electronic states at the VB edge of the Si NW, showing the eigenenergies of occupied (black) and unoccupied (gray) frontier electronic states with their DOS (highest occupied (HO) molecular orbital (MO) and the lowest unoccupied (LU) MO associated with Al). The VB edge of the Si NW is shown in blue, the MA state (β-LUMO) with its ionization energy of 0.136 eV is shown in red, defining the Fermi energy (dark yellow line). Iso-density plots of the MA state (β-LUMO) at a charge density of (c) 1 hole per Si atom (5 × 10^22^ cm^–3^, see to 1-nn O atom with DB adjacent to Al atom), (d) of one hole within the entire NW (1.27 × 10^20^ cm^–3^; 395 Si atoms), and (e) at the beginning of degenerate doping with a hole density equivalent to the VB DOS at *E*_V_, *N*_V_ = 1.02 × 10^19^ cm^–3^.^29^ Atom colors: Si NW atoms are cyan, Si atoms forming SiO_2_ are gray, O atoms are red, H atoms terminating outermost bonds are white. The black atom at the center-top of the NW is an Al atom; its 1-nn O atom with a DB is to its left.

Following the computational simulations, the proposed doping concept was experimentally validated through the implementation and demonstration of fully functional junctionless transistors. The two main factors to operate the device as a JLT are the minimized silicon thickness, to be able to deplete the channel completely in the off-state, and a contact that does not deteriorate carrier transport by introducing additional resistance, e.g., by a barrier at the contact interface. To this end, JLFETs were fabricated on a silicon-on-insulator (SOI) wafer having a final silicon channel thickness of 10 nm on top of a 20 nm thick buried silicon dioxide. The JLFET is operated by utilizing the body contact (substrate) as a back gate (BG) and employing platinum-silicide (PtSi) as source/drain (S/D) contacts, see Figure 2a,b. Devices consisting of a single NW and a NW array (10 NWs) were investigated, having a channel width of 50 nm (per NW) and with an effective channel length of the single NW (NW array) to be ∼700 nm (∼1.1 μm). The differences in the active channel length between the two devices arise from the different silicide lengths. It is noted that the back-bias will also have a certain influence on the contact barriers at the silicide-silicon interface, in addition to modulating the carriers in the channel.

**Figure 2:**
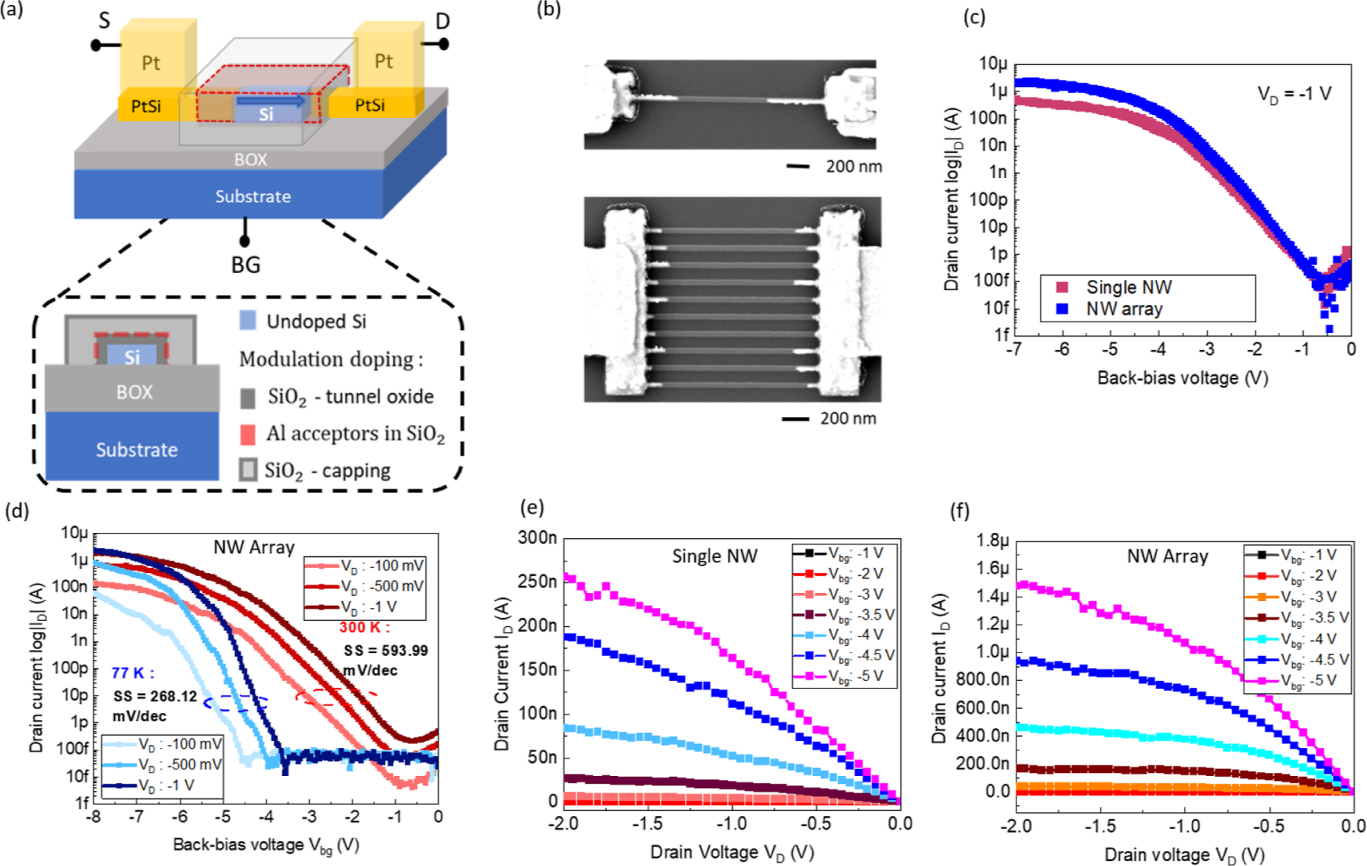
Modulation-doped Si NW JLFET. (a) Schematic of the back-bias controlled junctionless device, along with a cross-sectional view of the undoped Si channel that is modulation-doped. (b) Scanning electron microscope (SEM) images of a single NW and NW array (10 NWs) device. Transfer characteristics of (c) single NW JLFET, and NW array JLFET at room temperature. (d) Comparison of transfer characteristics at multiple *V*_D_ of an array JLFET measured at 300 and 77 K. Output characteristics of (e) single NW JLFET and (f) NW array JLFET.

The JLFET operates in three different regions: depletion, bulk conduction, and accumulation. Our devices operate across all three regions between 0 and −7 V applied to the back gate, having an equivalent oxide thickness (EOT) of 20 nm. Figure 2c shows the transfer characteristics of the realized JLFETs, which are clearly similar to a conventionally doped p-type JLFET.^9^ The high confinement of majority carriers within the nanowire allows the device to turn off even at *V*_bg_ = 0 *V* with an *I*_off_ in the range of 100 fA. The maximum on-current *I*_on_ at *V*_bg_(*V*_D_) = – 7 *V*(−1 *V*) is 463.5 nA (2.2 μA) for the single NW (NW array) device. This corresponds to a maximum *I*_on_/*I*_off_ ratio of 10^6^ (10^7^) for the single NW (NW array) device. However, the absolute *I*_on_ of the array device does not scale linearly with an increasing number of NWs, probably due to known differences in the channel lengths as attributed to the inhomogeneity of the contact silicidation process. The threshold voltage (*V*_th_) obtained from the transfer characteristics shown in Figure 2c for the single NW (NW array) device is about −2.71 V (−3.17 V) with negligible hysteresis. The subthreshold swing (SS), which quantifies the steepness of a device’s switching behavior, is evaluated next. A change in voltage of 486 mV (439 mV) is required to increase the current by 1 order of magnitude below the threshold for the single NW (NW array) device. The transistor characteristics of a different array device shown in Figure 2d demonstrate full device operation for temperatures down to 77 K without any degradation of the on-current. The stability of *I*_on_ as compared to 300 K can be attributed to higher mobility at lower temperatures. As expected from theory, the SS becomes lower with decreasing temperature due to the reduced thermal broadening of the Fermi–Dirac distribution near the Fermi energy.^33^ Please note that the thickness of the buried oxide acting as the gate oxide is a key limitation in approaching the thermal limit of SS in these devices. Please note that more competitive values for SS and V_TH_ will be achievable with gate-oxide thickness scaling and the design of a gate-all-around nanowire architecture, employing atomic layer deposited high-k dielectric passivation layers.

The output characteristics in Figure 2e,f describe how the drain current varies with drain voltage for different back-bias voltages. In analogy to traditional JLTs, the device operates like a simple gated resistor. The linear turn-on characteristics at a particular *V*_bg_ indicate the modified barrier properties of the modulation-doped device, which would otherwise show a sublinear behavior that is classically observed in undoped devices with a dominant Schottky characteristics.^34^ Thus, we conclude that the effect of both the source- and drain-side Schottky barriers at the PtSi/Si interface on the overall transport is minimized by the modulation doping process, favoring efficient carrier injection. The transport over these interfaces is analyzed in greater detail in the next section.

### Temperature Behavior of Transport Across Contact Interface

To understand the transport mechanism in this approach of externally doped Si nanochannels, we performed temperature-dependent measurements in a broad range from 250 to 385 K on the NW array device consisting of ten NWs to extract the barrier properties. The model is based on thermionic emission (TE) theory,^35,36^ and is investigated in temperature ranges where TE dominates. The temperature behavior of the device observed in the transfer and output characteristics is discussed in the first part, followed by insights into the contribution of the effective barrier under the given bias conditions to the overall transport.

Two prominent trends are observed in the transfer characteristics shown in Figure 3a with temperature change that are split into two different operating regimes: the off-state, and the active (saturation) state of the device. In the off-state, the gate voltage is adjusted to deplete the channel of the majority carriers, blocking current flow from the source to the drain. With increasing temperature, the device exhibits a broadening in the off-state current. Here, the remaining carriers in the channel are the thermally generated intrinsic carriers, which build a thermal barrier to the flow of holes from the source to the channel. As temperature increases, the number of thermally generated carriers increases, giving rise to an off-state leakage current.

**Figure 3:**
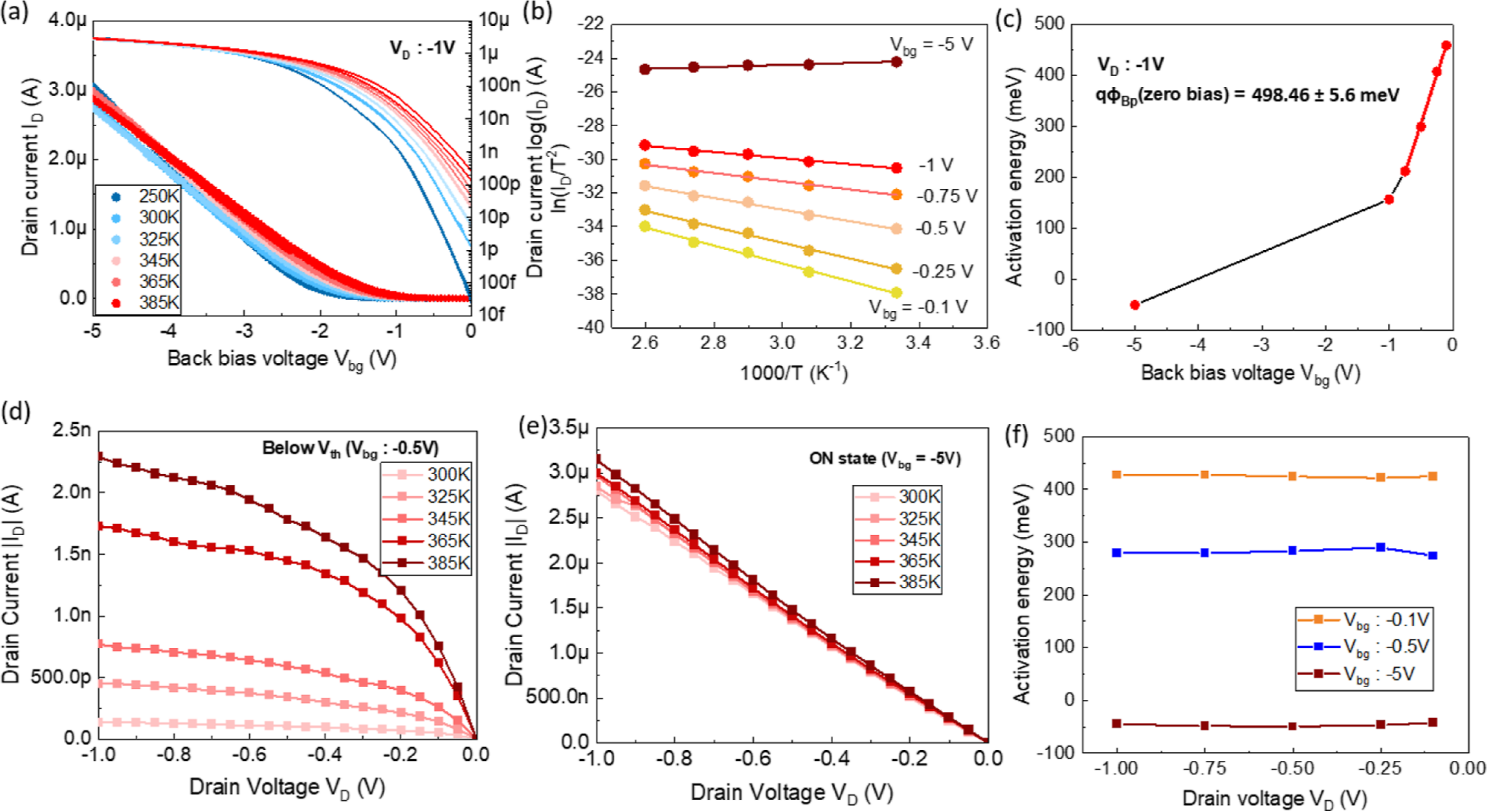
Temperature behavior and activation energies of NW array device. (a) Transfer characteristics of the array device at a *V*_D_ of −1 V measured at different temperatures in linear (left axis) and semilogarithmic (right axis) scale. (b) Arrhenius plot of the drain current for different back-bias voltages. (c) Activation energies extracted from the slopes of (b) as a function of back-bias voltage. Output current at different temperatures measured (d) at a *V*_bg_ of −0.5 V, and (e) at a *V*_bg_ of −5 V. (f) Activation energies plotted as a function of drain voltage for different back-bias voltages.

The active state of the device, where the saturation current is relatively constant (*V*_bg_ < *V*_th_, more negative back gate voltage than threshold voltage), serves to show the thermal dependence of the dominant transport mechanism at the metal–semiconductor interface. At room temperature, the Fermi-level alignment near the valence band would enhance the hole current with transport dominated by field emission or tunneling through the thin barrier. Additionally, if the concentration of holes induced in silicon by doping is high, the local Fermi level near the Si/SiO_2_ interface can shift notably into the VB DOS in analogy to a degenerately p-doped semiconductor (see also Figure 1e). The resulting 2D hole gas can further extend into the semiconductor, depending on the channel thickness, causing a modification of the electronic properties of the silicon channel. In reality, phonon scattering predominately decreases mobility at high temperatures, resulting in a lower on-current. For *T* > 300 K, a small decrease in the on-state *I*_D_ with a comparatively flatter slope can be observed on the linear scale. However, the relative change is less pronounced ( <0.5⁡μ A). In this situation, where the device is in its on-state, the channel resistance dominates carrier transport, with virtually no contribution due to increasing temperature, keeping the drain current fairly constant.

Next, the effective barriers^37,38^ are examined in three different operating regions: off-state, subthreshold region, and on-state, to confirm the transport behavior of the device. The effect of back-biased voltage in lowering the effective barrier can be seen in the activation energy plot with effective barriers extracted from transfer characteristics (Figure 3b,c). In addition, the influence of drain voltage in lowering the barrier can be visualized in the activation energies plotted from output characteristics (Figure 3d–f).

A natural barrier results for PtSi contacts aligned to p-Si due to a mismatch in the work functions of the metal and semiconductor. An effective barrier can be estimated at zero bias, closer to the natural barrier by extrapolating a linear fitting function through the effective barriers at low *V*_bg_ to zero. A value of ∼498 meV was obtained for the NW array device, which is closer to the literature values for PtSi contacts aligned to p-Si.^39–41^ When *V*_bg_ < 0 *V*, the effective barriers as plotted in Figure 3c come into play, with the onset of the bulk conduction at *V*_bg_ ≈ *V*_th_, followed by surface accumulation at large negative *V*_bg_. In the on-state, no physical barrier is present to hinder the transport, and the application of a pure thermionic emission model alone is not sufficient, implicating other mechanisms to dominate. Here the barrier width at the PtSi–Si interface is narrowed due to the modulation acceptor-doping induced field-effect that not just gates the contact barrier but also leads to a surface accumulation of charge carriers, enabling tunneling through the barrier and rendering the SBH largely irrelevant. Recent studies even suggest a positive effect of this quasi-opaque Schottky Barrier on the energy filtering of band tail states at ultralow temperatures.^42^ In our devices, when temperature changes, the saturation current is observed to remain nearly constant. Therefore, at *V*_bg_ = −5 *V*, mainly the tunneling transport mechanism dominates across the PtSi/Si contact interface, which is no longer temperature-dependent, as is indicated by the negative activation energy.

Figure 3d,e shows the output current measured below the threshold voltage, and in the on-state. Below *V*_th_, the temperature still plays a significant role due to the dominance of the barrier. In the on-state, the output current exhibits a linear behavior in the entire applied drain voltage range, indicating that the carriers can freely move without the need for any additional energy gain. Hence, the change in temperature in the on-state only marginally changes *I*_D_, and the carrier injection is independent of the natural barrier at sufficiently high *V*_bg_. The effective barriers are extracted from the measured output characteristics at different back-bias conditions, see Figure 3f. The change in *V*_D_ does not induce any additional barrier lowering, and the transport is mainly modified by the voltage at the back gate. This finding is supported by the constant effective barrier heights obtained from the Arrhenius plot. While limiting *I*_ON_ to a certain extent, such a constant barrier can act as an energy filter at ultralow temperatures and thus even help to further improve operation at deep-cryogenic temperatures.^42^ Thus, to fully understand the device’s operation toward cryogenic temperature conditions, it is essential to examine the exact influences on charge carrier transport properties at different temperatures, including carrier density and mobility.

### Conductivity, Carrier Density and Effective Mobility of the Modulation-Doped Channel

The carrier transport properties of the modulation-doped channel are studied by applying the transmission line model/transfer length method (TLM) on a specialized test structure. In accordance with TLM theory,^43,44^ the total resistance measured for different channel lengths is a linear function that relates to the metal/semiconductor contact resistance *R*_c_ and the semiconductor’s sheet resistance *R*_sh_, considering the metal’s resistance to be negligible. Such a TLM analysis performed on a 50 μM wide modulation-doped channel at 300 and 77 K is reported in Figure S2 in the Supporting Information In addition, the modulation-doped TLM structure is operated with an additional back-bias, as for the transistor device, to induce an electrostatic shift of the Fermi level and tune the carrier density of silicon in addition to the carriers induced by modulation doping.

Figure 4a,b shows a schematic illustration and the SEM image of the test structure. The total resistance obtained from each pair of contacts is plotted as a function of channel length for different back-biasing conditions to extrapolate the carrier properties such as conductance, carrier density, and field-effect (effective) mobility. This dependence can be quantified by relating the conductance (inverse of sheet resistance, *R*_sh_) to the product of the elementary charge, mobility, and density. The mobility obtained in this way is the overall field-effect mobility (μ_FE_), which approximates an effective mobility. The total density of carriers can be further split into gate-induced free carriers and doping-induced carriers (*p*_0_). Such a separation helps identify the carriers induced by modulation doping in the channel independent of the externally applied field (at *V*_*bg*_ = 0 V).^45,46^

**Figure 4:**
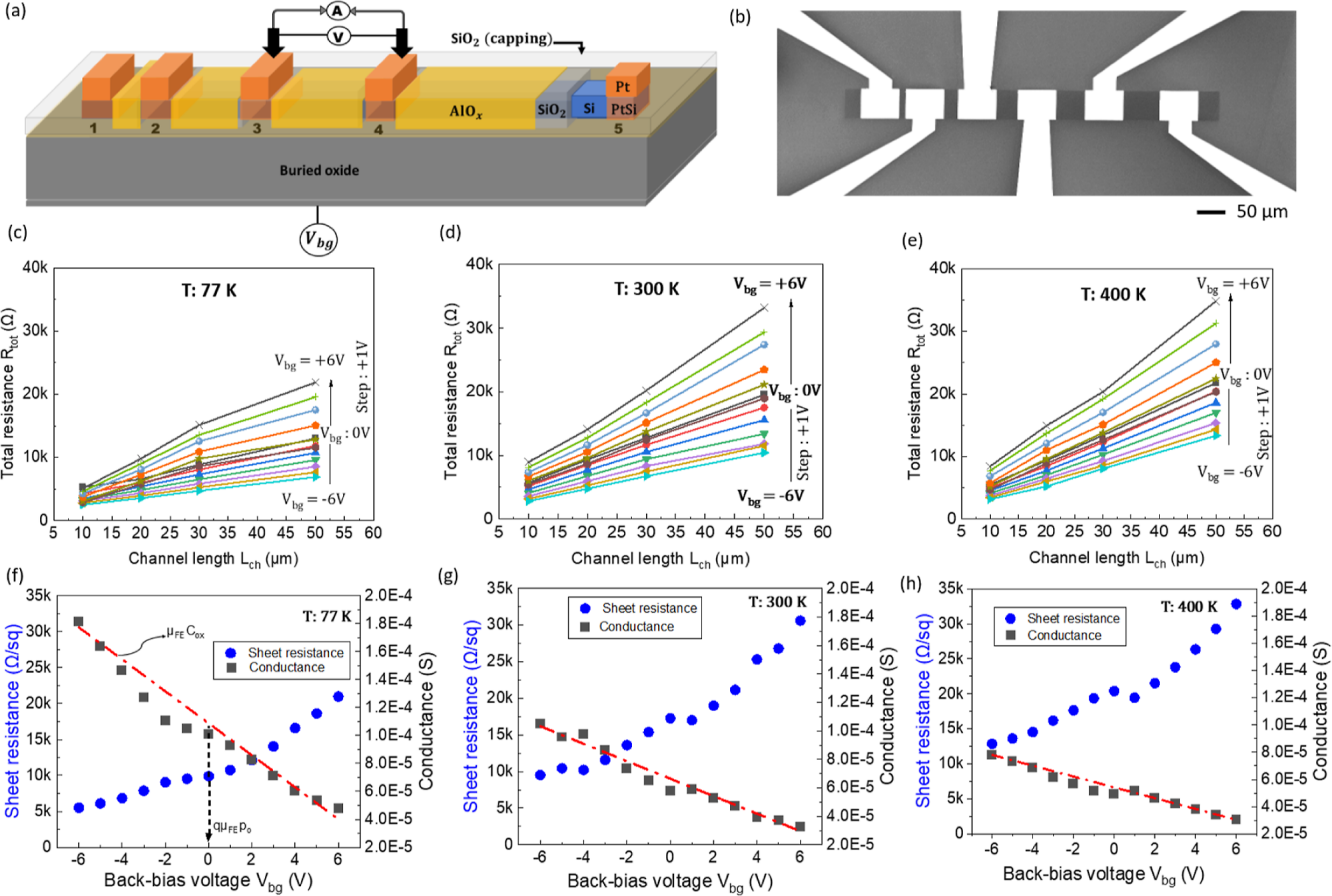
Back-biased TLM characterization. (a) Schematic of back-biased TLM structure showing contacts labeled from 1 to 5 set apart at different spacing. The different processed layers on the Si channel are shown between the contacts 4–5. (b) SEM image of the fabricated TLM structure. Plots of the temperature dependence of back-biased TLM for three different temperatures at (c) 77 K, (d) 300 K, and (e) 400 K. Plots of the corresponding sheet resistance and conductance extracted as a function of back-bias voltage at (f) 77 K, (g) 300 K, and (h) 400 K.

The TLM plot is reported for different back-bias voltages *V*_*bg*_ (+6 V to −6 V), where the dependence of the sheet resistance *R*_*sh*_ can be observed (Figure 4c–e). At *V*_bg_ = 0 *V*, the TLM plot exhibits a linear behavior, primarily due to the modulation doping process. The application of an external back-bias voltage further changes the slope. A higher slope is achieved for positive *V*_bg_, while a reduced slope is achieved for negative *V*_bg_. This behavior is mainly attributed to the type of dominant carriers in the channel. In undoped (Schottky barrier) FETs that are typically electrostatically doped by the gate, the device exhibits an ambipolar behavior with electron conduction for positive bias and hole conduction for negative bias.^35^ Such a device with equal barriers for holes and electrons would demonstrate nearly symmetric conduction. Contrarily, the investigated test structure results in an *R*_sh_ that is observed to increase monotonically with decreasing conductance toward positive *V*_bg_ (Figure 4f–h). A p-type characteristic is concluded from these indications. Table 1 gives a summary of all the parameters extracted using the back-biased TLM model. Further analysis of data at 300 K distinctly shows that modulation doping induced a high sheet carrier density of p-type carriers of $∼2.34\times 10^{12}cm^{-2}$ in the Si channel. A negative fixed charge density (*Q*_fix_) in the similar range of ∼−2 × 10^12^ cm^–2^ is also obtained from capacitance–voltage (CV) measurements (refer to Figure S3 in Supporting Information) on MOS capacitor structures, indicating the effect of modulation acceptor doping in the dielectric.

**Table 1:** Summary of Sheet Carrier Density, Volumetric Carrier Density, Field-Effect Mobility, and Contact Resistance Extracted at Different Temperatures From Back-Biased TLM. All *R* ^2^ are Larger than 0.99

temperature (K)	Sheet carrier density (cm^–2^)	volumetric carrier density (cm^–3^)	field-effect mobility (cm^2^V^–1^s^–1^)	specific contact resistivity (Ohm cm^2^)
77	2.05 × 10^12^	1.03 × 10^18^	331 ± 15	1.50 × 10^–5^
300	2.34 × 10^12^	1.17 × 10^18^	178 ± 7	1.15 × 10^–5^
400	2.95 × 10^12^	1.47 × 10^18^	115 ± 2	6.42 × 10^–6^

The temperature effects on mobility, mainly due to phonon and impurity scattering, would come into play with temperature changes. At room temperature and above, phonon scattering dominates the device mobility, which consequently results in a larger overall resistance, especially for longer channel lengths. With increasing temperature, a significant number of carrier–phonon interactions reduce the mean free path of carriers, leading to shorter ballistic transport. As the temperature decreases, this scattering is minimized due to reduced thermal energy, consequently increasing the ballistic mean free path of carriers and lowering the resistance of the channel. This can be visualized in Figure 4c from a reduced spread of resistance at 77 K, which increases correspondingly with increasing temperature (Figure 4d–e). More information about the extraction of the contact resistance can be found in the Supporting Information.

At lower temperatures (≤100 K), the contribution of ionized impurity scattering increases with higher dopant concentration in classical impurity-doped devices. Thus, their mobility drastically decreases due to these impurities that impede the motion of charge carriers, accounting for the significantly reduced mean free path. The main characteristic of modulation doping is a reduction of the latter phenomenon due to the relocation of dopants from the channel into the dielectric. Therefore, the mobility in a modulation-doped device increases with decreasing temperature. A mobility at 77 K of $∼331\pm 15cm^{2}V^{-1}s^{-1}$ is extracted with the sheet carrier density maintained stable at $∼2\times 10^{12}cm^{-2}$ . A nearly constant sheet resistance is also demonstrated at 77 and 300 K, see Supporting Information (Figure S2). The higher effective mobility at 77 K originates from a reduction of ionized impurity scattering, phonon freeze-out, and the externally applied voltage. When temperature increases, the mobility decreases to $∼178\pm 7cm^{2}V^{-1}s^{-1}$ at 300 K and $∼115\pm 2cm^{2}V^{-1}s^{-1}$ at 400 K, changing merely as an effect of increased phonon scattering. The differences in the obtained effective mobilities and the carrier mobilities reported earlier^5^ by Hall effect measurements arise from the respective evaluation models and how they account for the individual scattering events. As a result modulation acceptor doping yields a factor of roughly 2 higher hole mobilities in silicon than impurity doping at equal carrier densities. However, please note that the extracted effective mobility is an approximation that slightly overestimates carrier mobility due to the additional field-effect that accelerates carriers, though both experimental data sets follow similar trends, maintaining a nearly stable density and improving mobility with decreasing temperature, independent of the external voltage.

## Conclusions

The experimentally observed operation of p-type silicon junctionless nanowire transistors was reported without the need for direct impurity doping of the channel. The concept was enabled by moving acceptor-like Al-defects from the semiconductor into the surrounding SiO_2_ shell. The devices exhibit linear turn-on characteristics, temperature-stable on-state performance, and decreasing subthreshold slopes after cooling from 300 to 77 K. At higher temperatures, the transport mechanism in the on-state is almost independent of temperature, highlighting the device’s capability to operate reliably in a wide temperature range. A p-type characteristic is deduced from both the JLFET itself, as well as from TLM analysis, proving that the doping mechanism with Al-acceptors in SiO_2_ creates holes as majority carriers in silicon. The sheet density of carriers remains stable around 2 × 10^12^ cm^–2^ from 400 K down to 77 K, with the effective mobility increasing from 115 cm^2^V^–1^s^–1^ to 331 cm^2^V^–1^s^–1^. The disruptive modulation doping approach reveals that a junctionless transistor can be designed without the need for a highly impurity-doped channel, thereby aiming to eliminate all disadvantages associated with impurity doping, such as mobility degradation, statistical random dopant fluctuations, and scattering noise, simultaneously. The demonstrated JLFET concept thus provides a potential alternative to significantly overcome these key challenges at cryogenic temperatures. Hybrid-density-functional theory (h-DFT) calculations were carried out on silicon nanowire structures with 917 atoms, extending the upper end of the feasible size range for the employed solver. The results elucidated on the spatial-density relationship of the direct modulation acceptor states at the lower end of structure size, where this concept is still physically possible. In conclusion, there are no fundamental roadblocks to employing this doping concept in various nanoelectronic applications, particularly for energy-efficient cryogenic computing or as peripheral control electronics for quantum computing.

## Methods

### DFT Calculations

Real space calculations (nonperiodic boundary conditions) were carried out with a molecular orbital basis set (MO-BS) and Hartree–Fock (HF)/hybrid-DFT (h-DFT) methods, employing the Gaussian_09 program package^31^ with the GaussView program^47^ for visualization. Initially, the MO-BS wave function ensemble was tested and optimized for describing the energy minimum of the system (variational principle; stable = opt) with the HF method.^48–50^ Exact exchange interaction inherent to HF is crucial in obtaining accurate bond geometries (,^51^ Supporting Information). The Gaussian type 3-21G MO-BS^52^ was utilized. This HF/3-21G route was used for the structural optimization of approximants to obtain their most stable configuration (maximum integral over all bond energies). Achieved root-mean-square (RMS) and peak force convergences were 4.78 meV/*Å* and 72.8 meV/*Å* (26 and 396 μHa/a_B,0_), respectively. An own “bracket” code^53^ was applied, which intercepts the numerical noise during structural optimization, restarting the optimization with the most recent geometry. This “bracket” code slows down optimization jobs by 6% to 8%, but enables Gaussian to successfully optimize approximants with up to ca. 1500 atoms for a HF/3-21G computation route.^54^ Optimized geometries were used to calculate their electronic structure by testing and optimizing the MO-BS wave function ensemble with the nonlocal h-DF HSE06 after Heyd, Scuseria, and Ernzerhof with its 2006 parametrization.^55,56^ As MO-BS, the Gaussian_09 type 6-31G­(d) MO-BS was used, which contains d-polarization functions (HSE06/6-31G­(d)).^57^ For all calculations, tight convergence criteria were set to the self-consistent field routine, and no symmetry constraints to MOs were applied. Ultrafine integration grids were used throughout. Detailed accuracy assessments can be found in the Supporting Information of.^51,58,59^

### Fabrication Details

The devices and test structures were fabricated on an industrial-grade silicon-on-insulator (SOI) wafer, having a starting silicon film thickness of 12 nm on top of a 20 nm thick buried oxide with a resistivity of 9–18 Ω·cm. The nanowires are lithographically defined using a negative tone electron beam resist, hydrogen silsesquioxane (HSQ), which is employed as a hard mask for etching in the next stage. The 12 nm Si is rapidly etched in an inductively coupled plasma reactive-ion etching (ICP-RIE) tool using *SF*_6_ gas for a duration of 20 s. The resist is removed in a 1% HF solution to employ the doping process in the next stage.

The doping mechanism involves forming a stack of layers SiO_2_/AlO_*x*_/SiO_2_ precisely produced using different techniques, followed by an activation anneal. Initially, the nanowire is functionalized by rapid thermal oxidation (RTO, 900 °C) to form a tunnel oxide layer approximately 2.2 nm thick at the silicon surface. Following this, the aluminum atoms are incorporated as defects in the SiO_2_ lattice through 9 and 15 cycles of atomic layer deposition (ALD) of AlO_*x*_ using trimethylaluminum (TMA) and water vapor as precursors at a deposition temperature of 200 °C. The top SiO_2_ capping of 10 nm thickness is formed either ex-situ by plasma-enhanced chemical vapor deposition (PE-CVD, 350 °C, from SiH_4_and O_2_) or in situ using ALD (200 °C, tris­(dimethylamino)­silane and O_2_-plasma) to protect the functionalized surface. To form the Al-induced acceptors in SiO_2_ and to ionize them, a 30 s rapid thermal anneal at 850 °C in Ar-athmosphere is conducted. The processing of the modulation doping stack is done after structuring the silicon, which ensures a good areal coverage on the three exposed sides of the nanowire and provides a highly selective surface for doping. Next, the source and drain are defined by lithography steps using a bilayer positive tone resist (PMMA-MA/PMMA). To form the silicide contacts, the modulation doping stack is completely etched away in the contact regions using a 1% buffered HF solution containing 100 parts of 40% NH_4_F to one part of 38–40% HF, both in water. Platinum metal of 70 nm thickness is sputtered in a vacuum chamber at a coating rate of 1.5 Å/s with 2 nm titanium metal sputtered initially to improve adhesion. The metal is diffused into the silicon channels, forming a platinum-silicide alloy after an annealing is performed either in pure N_2_ or forming gas (90:10, N_2_: H_2_) atmosphere at 550 °C for 10 s. The process is highly reproducible as indicated by experiements on a large variety of transistor, TLM and Hall-bar test structures.

### Electrical Measurement Setup

The devices are measured in a vacuum cryo-station using a Keithley parameter analyzer. A 3-terminal model is set up to perform the measurements using source measurement units (SMUs). The TLM test structure is measured in pairs with one contact biased and the other unbiased, along with back-biasing. For the transistor’s transfer characteristics, the drain contact at a certain constant voltage is biased with the source contact held at 0 V. A body contact is enabled from the chuck where the voltage is swept from 0 V to −7 V in both forward and backward directions to monitor hysteresis. The output characteristics are then measured by sweeping the drain voltage at a constant back-bias voltage. Temperature-dependent measurements were performed in the same tool using liquid N_2_ to cool down to 77 K, and warm-up using a heat control unit.

## Supplementary Material





## Notes

Additional theory of modulation acceptor doping; schematic representation of the fabrication process flow; density functional theory (DFT) video short description; transfer length measurement analysis on TLM structure without back-biasing; evaluation of contact resistance; modulation-doped MOS capacitor (PDF)
Supporting video material showcases the effect of modulation doping as calculated by DFT (MP4)
