# Supplementary material for: Junctionless Silicon Nanowire Transistors without the Use of Impurity Doping
Source: ACS Nano. 2026 Feb 23;20(9):7508–17. doi: 10.1021/acsnano.5c17282 (PMC12981024; doi:10.1021/acsnano.5c17282)
Supplement: Supplementary file 1 [file nn5c17282_si_001.pdf]

Supporting Information

Junctionless Silicon Nanowire Transistors without the Use of Impurity Doping

Soundarya Nagarajan[1,2], Dirk König[3], Ingmar Ratschinski[4], Giulio Galderisi[1,2], Somayeh Shams[4], Thomas Mikolajick[1,2], Daniel Hiller[4], and Jens Trommer[1]

[1]NaMLab gGmbH, Nöthnitzer Str. 64a, 01187 Dresden, Germany

[2]Institute of Semicondutors and Microsystems, Technische Universität Dresden, Nöthnitzer Str. 64, 01187 Dresden, Germany

[3]Department of Material Physics, Research School of Physics, Australian National University (ANU) Canberra, ACT 2601, Australia

[4]Institute of Applied Physics (IAP), Technische Universität Bergakademie Freiberg, Leipziger Str. 23, 09599 Freiberg, Germany

Outline:

Page 2 … 1. Theory of Modulation Acceptor Doping
Page 3…..2. Schematic of Fabrication Process
Page 3 … 3. Density Functional Theory (DFT) Video Short Description
Page 4 … 4. Transfer length measurement analysis on TLM structure without back-biasing
Page 5…. 5. Evaluation of the Contact Resitance
Page 6 … 6. Modulation-doped MOS capacitor
Page 8 … References

1. Theory of Modulation Acceptor Doping

Generally, modulation acceptor (MA) states in SiO_2 for adjacent Si are constituted by a suitable chemical element of the III. main group (IIIA: Al, Ga, In) or the III. auxiliary group (IIIB, transition metals: Sc, Y, La) replacing Si in SiO_2 in compound with the dangling bond (DB) on one of the four 1st next neighbour (1-nn) O atoms [R1]. This O-DB is the state which can localize an extrinsic electron from adjacent Si, and the group IIIA/IIIB element replacing Si provides the positive charge background to facilitate the localization of an extrinsic electron at the O-DB. Such a trivalent atom replacing Si is positively ionized by its remaining three 1-nn O atoms to each of which it still has one chemical bond. This ionization is highest for group IIIA/IIIB elements having a possibly small ionization energy E_{ion}, and a possibly low electronegativity (EN), both maximizing the positive ionization of the substitutional atom. To this end, Al, Ga, and in particular Sc are the best substitutional impurities for Si in terms of providing a maximum relaxation energy E_{relax} to the O-DB into which initially fully occupied VB states at the VB edge energy E_V of Si can provide an electron, leaving behind a free hole in the VB DOS [R2]. A second-order parameter for E_{relax} is the difference in ionic radius r_{ion} between the nominal Si atom and the group IIIA/IIIB atom replacing Si. Differences in r_{ion} of > 25 % relative to the nominal Si atom result in a notable reduction of E_{relax} due to local stress and delocalization of the MA state [S1] as occurring for Si in SiO_2 being substituted with Y or La.

2. Schematic of Fabrication Process

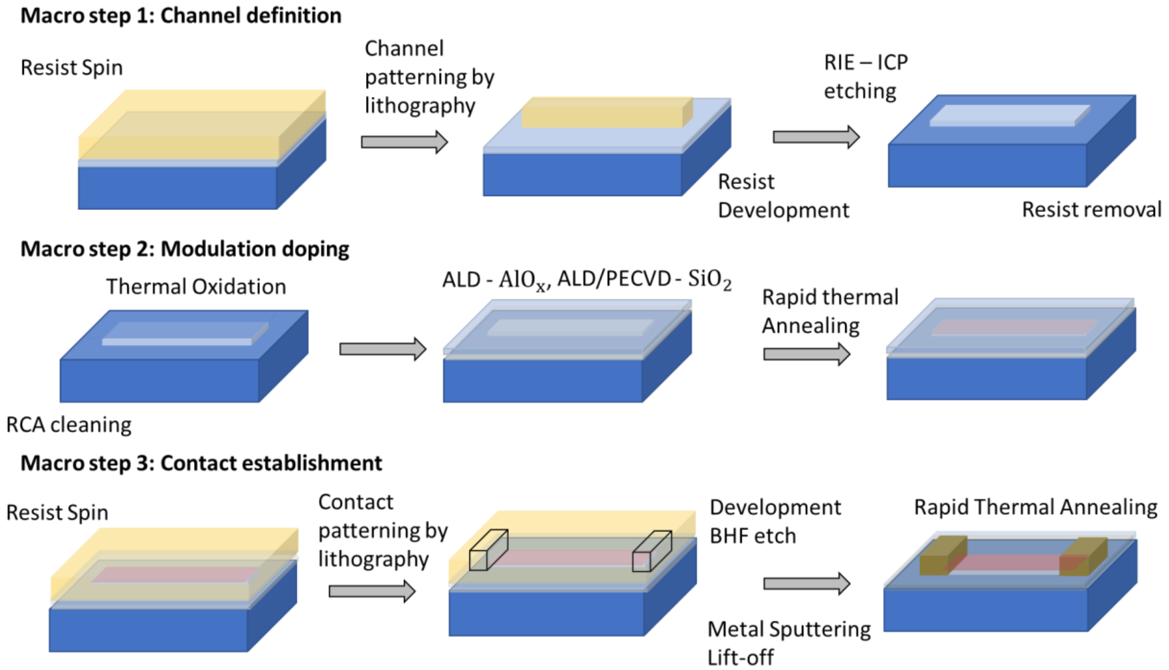

Figure S1: Process route followed for fabricating JLFETs and test structures described in the main manuscript.

3. Density Functional Theory (DFT) Video Short Description

Related to Figure 1c-e of the main manuscript the below named supporting video material showcases the effect of modulation doping as calculated by DFT.

DFTSi395-2ML.SiO2.blkAl_HSE06.631Gd_s324289_bLUMO4887sq_1e18to5e22cm-3.mp4

4. Transfer length measurement analysis on TLM structure without back-biasing

The current-voltage (IV) characteristics measured between different adjacent contact pairs are analysed to obtain the total resistance of each segment. By plotting the total resistance as a function of the channel length, the sheet resistance and specific contact resistivity are inferred from the slope and intercept of the linear fitting function respectively (see Fig. 7(a-b)) [S3-S4]. A R_{sh} of 25.18 k ± 1.914 kΩ/Sq is obtained at 300 K with a contact resistivity ρ_C of $1.975 \times 10^{-5} \pm 6.8 \times 10^{-6}$ Ω·cm^2. At 77 K, nearly the same R_{sh} of ~ 27.08 k ± 2.55 kΩ/Sq with a slightly lower contact resistivity ($3.925 \times 10^{-6} \pm 9.12 \times 10^{-6}$ Ω·cm^2) is obtained. This behaviour of an unchanged sheet resistance and a lower specific contact resistivity can be explained by the properties of the modulation doped channel and the transport mechanism across the metal silicide-silicon interface.

The constant sheet resistance is mainly favoured by the fact that the generation of carriers in the modulation doped devices is independent of the thermal energy. In addition, the scattering events (due to ionized impurities, lattice vibrations, and interface roughness) are completely suppressed at 77 K, which allows for a scattering-free carrier transport within the channel. Moreover, thermionic emission becomes negligible at temperatures as low as 77 K. Therefore, the carrier injection across the barrier is controlled via tunneling mechanism, leading to an even reduced contact resistivity. The behaviour of the test structure is expected to remain relatively stable in a highly optimized fabrication process, such as in our case achieved by the use of different techniques to precisely produce the modulation doping layers and performing additional annealing treatment to prevent surface recombination activity.

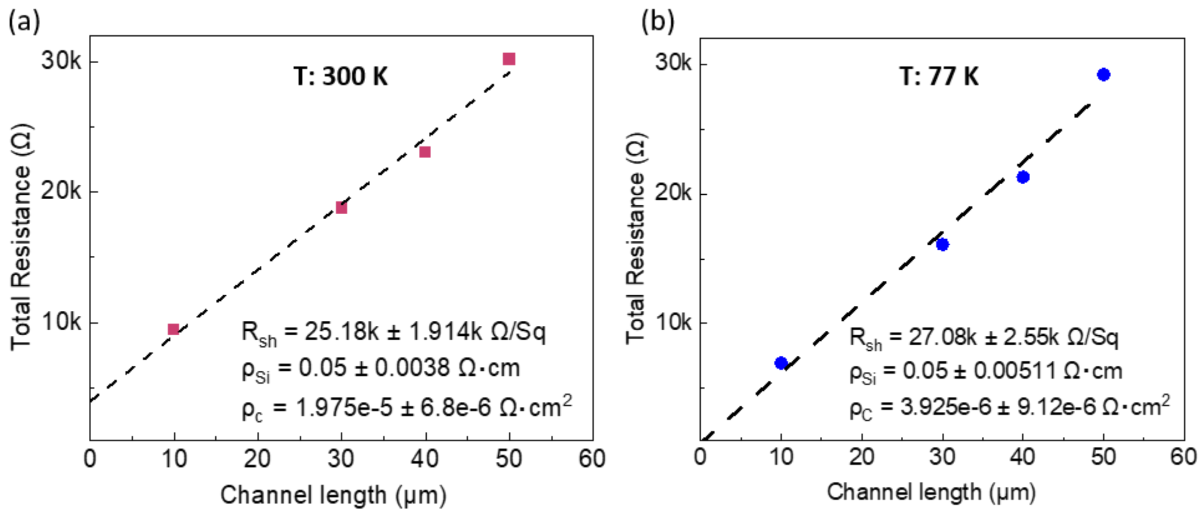

Figure S2: Transfer length measurements. TLM plot of the investigated structure at a) 300 K, and b) 77 K without any back-biasing reported along with extracted parameters

5. Evaluation of the Contact Resitance

The specific contact resistivities were obtained from the TLM measurement at three different temperatures without the influence of the back-bias voltage (at $\boldsymbol{V_{bg} = 0V}$). The intercept at the y-axis of the TLM plot (See Fig 4 (c-e) in main manuscript) at $\boldsymbol{V_{bg} = 0V}$ gives twice the contact resistance. This contact resistance multiplied by the area of the contact gives the specific contact resistivity, which are summarized in Table 1. With decreasing temperature (down to 77 K), the specific contact resistivity remains greatly stable compared to RT, indicating that the dominant conduction mechanism is independent of temperature. This behavior is attributed to the well-known tunneling (field emission) mechanism. In contrast, as the temperature increases to 400 K, the specific contact resistivity decreases following thermionic emission behavior. This dependance can be explained from the below equation where the barrier height (qϕB) and temperature T relates to the contact resistivity [S5]:

$$\boldsymbol{\rho_C = \frac{k_B}{qA^*T} exp\left(\frac{q\phi_B}{k_B T}\right)}$$

These results shed light on the underlying transport mechanism, which transitions from thermionic emission at high temperatures to tunneling with decreasing temperature. This characteristic response can be realized only in highly engineered contacts such as fabricated for this work using high work function metallic-silicide contacts and annealing treatments. If not optimized, a decrease in temperature can modify the contact behavior, making it Schottky-like and increasing the barrier-limited behavior [S6]. This is not the case here, as the obtained contact resistivities for the investigated temperature range (77 K – 400 K) are very low and greatly comparable to that of a good ohmic contact [S7].

Table S1: Summary of specific contact resistivity obtained from TLM measurements at three different temperatures at $\boldsymbol{V_{bg} = 0V}$.

Temperature (K)	Specific Contact Resistivity (Ω· $\boldsymbol{cm^2}$)
77	1.50E-05
300	1.15E-05
400	6.42E-06

6. Modulation-doped MOS capacitor

A MOS capacitor structure is studied by capacitance-voltage (C-V) measurements to determine the effect of modulation acceptor doping on the dielectric. The same stack structure as for the junctionless back gate transistor and back gated TLM devices is used, i.e., a monolayer of ALD-AlO_x sandwiched between a thermally grown tunnel oxide (~2.2 nm) and an ALD-grown SiO_2 capping layer (~10 nm). A schematic cross-section of the MOS capacitor structure with thermally evaporated aluminum contacts is shown in the lower left inset of Figure S2. The C-V curves exhibit a flat-band voltage shift (ΔV_{FB}) caused by ionized Al-induced acceptor states in modulation doped SiO_2:Al samples (blue) in comparison to pure SiO_2 (grey). The extracted fixed charge densities (Q_{fix}) are shown in the upper right inset: where the reference samples made of pure SiO_2, i.e., without ALD-AlO_x (grey) have a positive Q_{fix} of about $+1\times10^{12}$ cm^{-2} (as conventionally observed for SiO_2), and the SiO_2:Al samples have a negative Q_{fix} of about -2×10^{12} cm^{-2}. Hence, the Al-acceptor states in SiO_2 led to a ΔQ_{fix} of ~3×10^{12} cm^{-2} by capturing electrons from the adjacent silicon volume. This compares reasonably well with the sheet hole density extracted from the back gated TLM structures at room temperature of 2.34×10^{12} cm^{-2} (cf. Table 1 in the main text). In order to explain any discrepancy between ΔQ_{fix} and the sheet carrier density, we want to point out that also an interaction of the Al-induced acceptor states with dangling bond defects at the Si/SiO_2 interface was previously observed [S8]. Accordingly, singly occupied (P_b-type) dangling bonds defects, which represent the interface trap level density (D_{it}), can be discharged by the acceptor states, which is considered as an alternative source of electrons to ionize acceptors and to generate negative Q_{fix} but without creating holes as majority carriers in the Si volume. The exact details of these two modulation acceptor ionization processes are not yet fully understood. However, it can be concluded that modulation acceptor doping is possibly beneficial for the interface defect density, which is supported by the slightly reduced peak height of the conductance-voltage curves (dotted lines in Figure S2) and the extracted D_{it} values of the SiO_2:Al sample (~5.3×10^{11} cm^{-2}eV^{-1}) vs. the SiO_2 reference sample (~3.6×10^{11} cm^{-2}eV^{-1}). The achieved fixed charge density is further dependent on the tunnel-SiO_2 thickness.

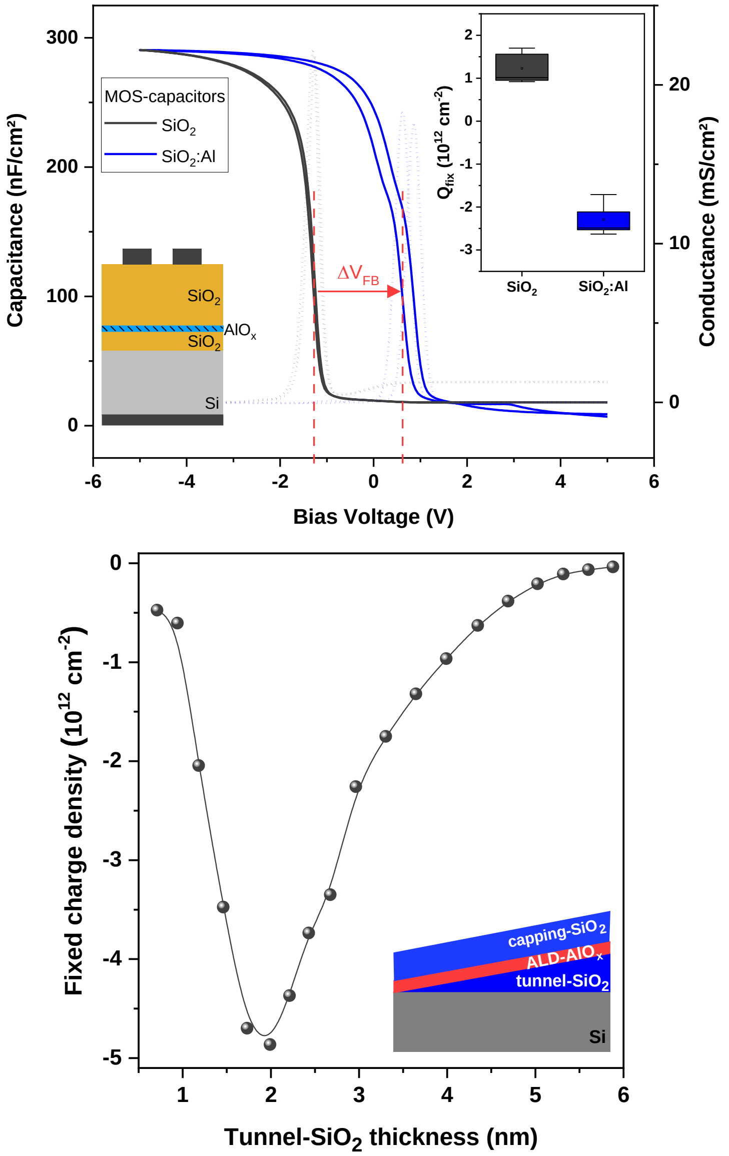

Figure S3: a) High-frequency capacitance-voltage curves of MOS capacitors (schematically shown in the lower left inset) to demonstrate the flat-band voltage shift caused by Al-induced acceptor states in SiO_2 in modulation doped SiO_2:Al samples (blue) in comparison to pure SiO_2 (grey). The extracted fixed charge densities are shown in the upper right inset. The associated conductance-voltage curves (right axis) are shown as dotted lines. b) Effective negative fixed charge density of Al-induced acceptor states in SiO2 measured as a function of tunnel-SiO2 thickness using MOS-capacitors. The tunnel-SiO2 thickness variation was realized by slant-etching. Here the ALD-AlO_x layer comprises of only 15 ALD-cycles, the capping layer thickness is 10 nm. A schematic cross-section of the sample is shown in the inset. The solid line (spline) is just a guide to the eye. The plot verifies that the maximum fixed charge densities, which are equivalent to highest Al-acceptor state ionization densities, are achieved for tunnel-SiO2 thicknesses of approximately 2 ± 0.5 nm.

References

[S1] König, D., Hiller, D., & Smith, S. SiO2 Modulation Doping for Si: Acceptor Candidates. *Physical Review Applied*, *10*(5), 054034, (2018).

[S2] König, D., Hiller, D., Gutsch, S., Zacharias, M., & Smith, S. Modulation doping of silicon using aluminium-induced acceptor states in silicon dioxide. *Scientific reports*, *7*(1), 46703, (2017).

[S3] Grover, S., Sahu, S., Zhang, P., Davis, K. O., & Kurinec, S. K. Standardization of specific contact resistivity measurements using transmission line model (TLM). In *2020 IEEE 33rd International Conference on Microelectronic Test Structures (ICMTS)* (pp. 1-6). IEEE, (2020, May).

[S4] Vinod, P. N. Specific contact resistance measurements of the screen-printed Ag thick film contacts in the silicon solar cells by three-point probe methodology and TLM method. *Journal* of Materials Science: Materials in Electronics, 22, 1248-1257, (2011).

[S5] Sze, S. M., Li, Y. & Ng, K. K. Physics of semiconductor devices *(John wiley & sons*, 2021).

[S6] Nagarajan, Soundarya, et al. "Analyzing Carrier Density and Hall Mobility in Impurity-Free Silicon Virtually Doped by External Defect Placement." *Advanced Functional Materials* 35.7 (2025): 2415230 (Supporting information).

[S7] Stavitski, Natalie, et al. "Evaluation of transmission line model structures for silicide-to-silicon specific contact resistance extraction." *IEEE transactions on electron devices* 55.5 (2008): 1170-1176.

[S8] Hiller, D., Jordan, P. M., Ding, K., Pomaska, M., Mikolajick, T., & König, D. Deactivation of silicon surface states by Al-induced acceptor states from Al–O monolayers in SiO2. *Journal of applied physics*, *125*(1), (2019).
